# Mental Health Literacy and Information Needs of Young Adults With First Episode Psychosis and Their Support Persons

**DOI:** 10.1111/eip.70148

**Published:** 2026-03-06

**Authors:** Colleen Murphy, Madison P. Hardman, Kristin A. Reynolds, Natalie Mota

**Affiliations:** ^1^ Department of Clinical Health Psychology University of Manitoba Winnipeg Manitoba Canada; ^2^ Department of Psychology University of Manitoba Winnipeg Manitoba Canada; ^3^ Department of Psychiatry University of Manitoba Winnipeg Manitoba Canada

**Keywords:** early psychosis intervention, first episode psychosis, information needs, mental health literacy, support persons

## Abstract

**Background:**

First episode psychosis is often poorly recognised at onset despite significant impacts on the young person and their support persons. Higher mental health literacy may improve symptom recognition, access to treatment, and perceptions of recovery. This research evaluated the mental health literacy and information needs of young adults with first episode psychosis and their support persons.

**Methods:**

Participants were recruited from a first episode psychosis clinic in Canada and included 57 service users and 43 support persons (*N* = 100). Data were collected through an online mixed‐methods survey. Linear and logistic regressions examined the relationships between treatment duration, outcome, mental health literacy, and information needs. Specific information needs were identified through open‐ended questions and reviewed using content analysis.

**Results:**

Both participant groups reported a broad range of mental health literacy and information preferences. Longer treatment involvement significantly predicted greater willingness to seek out information. Higher mental health literacy significantly predicted higher treatment satisfaction among service users and support persons, and lower stress levels among service users. Participants identified additional information needs related to *Diagnosis and Recovery, Treatment, and Healthy Functioning;* and felt it was important to learn information through *Reliable Sources, Group Programming,* and *Peer Support*.

**Conclusions:**

Findings highlight the important role that mental health literacy can play in treatment satisfaction for service users and their support persons, and provide a better understanding of their information needs. These findings have implications for psychoeducation and treatment planning in first episode psychosis.

## 1 Introduction

First episode psychosis is a serious medical condition associated with hallucinations, delusions, grossly disorganised behaviour and thoughts, and negative symptoms (Iyer et al. 2015; Puntis et al. 2020). The condition is also associated with cognitive impairment and significant disruption to socio‐occupational and academic functioning (Barder et al. 2013; Stouten et al. 2017). Onset of symptoms typically occur during late adolescence or early adulthood (Kirkbride et al. 2017). During this critical developmental period, most young person's continue to reside in the family home, and the impact of psychosis can extend to the individual's family and support persons. Indeed, families often provide a significant amount of support to the young person (Addington et al. 2001; Jansen et al. 2015) and play a key role in early psychosis intervention (EPI) services (Addington et al. 2005). However, caregiving responsibilities can lead to increased parental stress and poorer well‐being (Jones 2009; Lowenstein et al. 2010; Martens and Addington 2001; Sin et al. 2005).

### 1.1 Mental Health Literacy (MHL) in First Episode Psychosis

MHL is defined as the knowledge and beliefs about mental disorders and how these factors impact the recognition, management, and prevention of such disorders (Jorm 2012). According to health literacy theory, recognising that one has an illness, and acknowledging one's experience or change in experiences as evidence of symptoms, is one of the earliest and most important phases of the help‐seeking process. Moreover, poor MHL has been found to negatively impact the treatment‐seeking process and the individual's satisfaction with treatment, serving as a barrier to accessing mental health treatment (Smith and Shochet 2011; Xu et al. 2018).

The limited research examining MHL and psychosis in the general population demonstrates that most people have a poor understanding and recognition of psychotic symptoms, as well as poor understanding of psychosis management, treatment options, and prognosis (Reavley and Jorm 2011). One study found that only 23% of the general Canadian population reported knowledge of the term, ‘psychosis’ (Addington et al. 2012). The lack of information presents a significant barrier to early identification and recovery in first episode psychosis (Jackson and McGorry 2009). Due to these gaps in MHL, the young person and their support persons might not recognise the early warning signs or symptoms of psychosis. Past studies, including those that employed qualitative analyses, have shown that the observed changes in the young person can be attributed to other stressors (Al Taher and Fox 2023), a developmental stage (Addington and Burnett 2004; Judge et al. 2008), or a less stigmatising medical condition such as depression (Czuchta and McCay 2001; Franz et al. 2010; Judge et al. 2008). Once the need for help is recognised, there are challenges navigating the mental health system and accessing services (Addington et al. 2012). These delays in help‐seeking behaviour are of particular concern due to the well‐established relationship between the duration of untreated psychosis (DUP) and treatment prognosis (Perkins et al. 2005). Among those who were diagnosed with a first episode psychosis, MHL was found to have a significant effect on DUP independent of education and immigration history (Takizawa et al. 2021).

Specialised EPI teams were developed to provide early detection and accessible community‐based assertive care that includes pharmaceutical intervention, case management, psychoeducation, and psychosocial rehabilitation. Compared to standard community mental health care, EPI services are more beneficial in terms of reducing symptoms, improving functioning, reducing disengagement, and reducing psychiatric hospitalizations (Harvey et al. 2007; McGorry 2015; Puntis et al. 2020; Randall et al. 2015). While prior research has shown that the general population has a poor understanding of psychosis, no research to date has examined the MHL of service users with first episode psychosis involved in an EPI service. Given that psychoeducation is a cornerstone of EPI, it also remains unclear whether MHL and information needs change over the time that the service user is receiving treatment, as has been found among patients with cancer (Matsuyama et al. 2013).

### 1.2 Information Needs and First Episode Psychosis

Information preferences has been examined in patients with medical conditions, such as patients with inflammatory bowel disease who have been recently diagnosed (Bernstein et al. 2011) and those with longstanding disease (Wong et al. 2012); in patients with mental health conditions such as anxiety (Bernstein et al. 2022), child anxiety (Mak et al. 2014), and depression (Bernstein et al. 2017); and in specific populations such as post‐secondary students (Stewart et al. 2014) and older adults (Reynolds et al. 2023). It has yet to be examined in first episode psychosis. Moreover, qualitative studies have found that, even after an initial consultation or the initiation of pharmacological treatment, young persons with first episode psychosis and their support persons have unmet information needs that cause significant distress (McKenzie 2006; Sin et al. 2012). This finding is of particular concern, as lack of information related to medical care is associated with increased confusion, decreased compliance and satisfaction with treatment, and increased likelihood of symptom relapse (Hall et al. 1988).

Given that family and other support persons are often heavily involved in EPI services, it is conceivable that they might have their own information needs that differ from the young person with psychosis. For example, Parker et al. (2007) reviewed studies examining the differences in information needs among patients with cancer and their caregivers. While both groups had high information needs at the different stages of illness, there were also important differences between the groups; specifically, patients wanted less information while caregivers wanted more information near the end‐stage of the illness. To date, no known research has examined whether differences in information needs exist between service users involved in an EPI service and their support persons.

### 1.3 Objectives and Hypotheses

Using a mixed‐methods approach, the primary aim of the present study was to evaluate the MHL and information needs of service users involved in an EPI service and of their designated support persons. Within this aim, we identified five objectives within the present study: (1) to describe the MHL and information needs of service users and their support persons; (3) to explore whether MHL and information needs differ by length of involvement with an EPI service; (4) to examine whether MHL is related to more positive attitudes toward treatment and improved treatment outcomes; and (5) to ascertain unexplored information needs identified by the sample.

## 2 Method

### 2.1 Participants

Participants were recruited from an EPI service located in an urban setting in Central Canada. The EPI service is publicly funded through the provincial health system and provides outpatient, community‐based care for a period of 2 years to individuals who are 13–35 years of age and have experienced a first episode of psychosis. The service strives to provide early identification and rapid access to care, with approximately 100–115 service users involved in the programme at any one time. Service users receive ongoing follow‐up and care provided by a mental health clinician (i.e., nurse, social worker, occupational therapist) and psychiatrist, including medication management, case management, supportive counselling and education, and family support and education. Service users are invited to attend an 8‐session manualised psychoeducational group (2×/year), recreation group (weekly), and other evidence‐based psychological interventions (i.e., cognitive behavioural therapy for psychosis, cognitive remediation, internalised stigma group) as well as access to psychological assessment. Support persons are invited to attend a 5‐week manualised psychoeducational group (2×/year).

For this study, there were two groups of participants: (1) service users aged 18 years or older (*M* = 24.08; SD = 4.02) involved with the EPI service, and (2) a designated support person aged 18 years or older (*M* = 48.32; SD = 11.74) who was identified by the service user. One support person per service user was permitted to complete the survey. The service user could participate in the study even if their support person did not and vice versa. We aimed to recruit 100 participants from each participant group (*n* = 200) to detect a medium effect size (power = 0.80, α = 0.05).

### 2.2 Procedure

Data were collected between December 2021 and February 2023. Participants were recruited on a rolling basis, initially through recruitment posters and invitations from their mental health clinicians to complete the survey. However, the survey was launched when COVID‐19 public health orders were in place; thus, in‐person visits at the EPI clinic were limited and all appointments (i.e., in‐person and virtual) were primarily focused on the service users' well‐being. Psychoeducational and psychological group interventions were strictly virtual. These processes led to reduced contact with support persons, as they were less likely to accompany service users to in‐person appointments or join virtual appointments. Furthermore, there were challenges with the follow‐through with some participants, whereby they would indicate interest when initially invited to participate, but were less likely to complete the survey at home when they received the unique link via email. These issues led to poor uptake, and as such, additional recruitment strategies were introduced. These strategies including extending the data collection period, mailing invitations, and providing immediate access to the survey via computer tablets so participants could complete the survey while on‐site.

Two versions of the survey were developed using Qualtrics: one for service users and one for their designated support persons. Prior to distribution, the surveys were reviewed by co‐authors with expertise in MHL and psychosis, the EPI team, a young adult and their family member who were previously involved with the EPI service, and peer‐support community organisations and groups for persons with lived experience of psychosis and their family members. Surveys were revised as necessary based on reviewer feedback. Both versions of the survey achieved a rating equivalent to a Grade 8.5 reading level using the Flesch–Kincaid test, which is only slightly above the recommended score range of 7.0–8.0 (Microsoft Cooperation 2019). Participants received a $10 gift card as a thank‐you for their time. This project was approved by the University of Manitoba Bannatyne Campus Health Research Ethics Board (HS23765) and the Shared Health Research Impact Committee (RI2020:031). All participants provided informed consent to participate.

### 2.3 Measures

#### 2.3.1 Objectives 1 and 2

##### 2.3.1.1 MHL

Knowledge and beliefs of psychosis was assessed using a revised version of the MHL Scales (Reavley et al. 2014). The original version of these scales demonstrated good construct validity in past research (Reavley et al. 2014; Wei et al. 2015). Using a 9‐point Likert scale, participants rated their knowledge of psychosis terminology and treatment services (*Poor* = 0, *Very good* = 8, *Prefer not to respond* = 9), as well as their understanding of their role in their own or their loved one's recovery, respectively (*Not at all* = 0, *Completely* = 8, *Prefer not to respond* = 9). Using this latter scale, service users also rated their understanding of their family's role within the EPI service. Finally, service users and support persons rated their agreement to three statements that reflected negative stereotyped beliefs about psychosis (e.g., persons with psychosis are violent, weak‐minded, or have a life‐long illness) (*Not at all* = 0, *Completely* = 8, *Prefer not to respond* = 9).

##### 2.3.1.2 Information Needs

The Mobilising Minds Research Group developed questions to evaluate information needs used in prior research. First, participants were asked if they had used the Internet to search for information about psychosis. Second, using a 9‐point Likert scale, participants were asked to rate (a) their familiarity with psychosis treatments (*Not at all familiar* = 0, *Very familiar* = 8, *Prefer not to respond* = 9), (b) the helpfulness of various resources (*Not at all helpful* = 0, *Very helpful* = 8, *Prefer not to respond* = 9), and preferences related to specific (c) types of treatment information (*Not at all important* = 0, *Very important* = 8, *Prefer not to respond* = 9), (d) sources of information (*Not at all likely* = 0, *Very likely* = 8, *Prefer not to respond* = 9), and (e) formats of delivery (*Not at all preferred* = 0, *Very much preferred* = 8, *Prefer not to respond* = 9). Participants' preferred amount of information was assessed using a 6‐point Likert scale (*0 pages* = 0, *2 pages* = 1, *4 pages* = 2, *6 pages* = 3, *8 pages* = 4, *10 or more page*s = 5, *Prefer not to respond =* 6). Total information needs scores for each subscale were calculated by summing items on each scale.

#### 2.3.2 Objective 3

##### 2.3.2.1 Duration of Involvement With EPI

Both service users and support persons were asked to estimate the number of months the service user had been involved with the EPI service, which were then corroborated with the service user's medical chart.

#### 2.3.3 Objective 4

##### 2.3.3.1 Attitudes Towards Seeking Psychological Help

The 10‐item Attitudes Towards Seeking Professional Psychological Help Short‐Form (ATSPPH‐SF) scale (Fischer and Farina 1995) measures attitudes towards mental health treatment using a 4‐point rating scale (*Disagree* = 0, *Agree* = 3). Responses on items 2, 4, 8, 9 and 10 are reverse scored and all items are summed to generate a total score (0–30), with higher scores representing more favourable attitudes towards psychological treatment. The ATSPPH‐SF has adequate internal consistency (α = 0.77–0.78) and good test–retest reliability (*r* = 0.80) (Elhai et al. 2008; Fischer and Farina 1995).

##### 2.3.3.2 Perceived Stress

The Kessler Psychological Distress 6‐Item (K6) is a self‐report measure that asks about how the respondent has been feeling over the past 30 days using a 5‐point Likert scale (*None of the time* = 0, *All of the time* = 4) (Kessler et al. 2002). Scores on all items are summed to generate a total score (0–24), with higher scores indicating greater distress. A cut point of ≥ 5 on the K6 identifies those with moderate mental distress (Prochaska et al. 2012). Previous research assessing the psychometric properties of this measure (Kessler et al. 2002; Umucu et al. 2022) has demonstrated good internal consistency (α = 0.86–0.89).

##### 2.3.3.3 Client Satisfaction

Satisfaction with the EPI service was assessed using the Client Satisfaction Questionnaire 8‐item (CSQ‐8) version (Larsen et al. 1979). Participants responded to each item using a 5‐point Likert scale with unique response options for each question. Scores on items 1, 3, 6 and 7 are reverse scored, and then all items are summed to generate a total score. Total scores range from 8 to 32, with higher scores indicating more positive experiences (i.e., 8–20 = low satisfaction, 21–26 = medium satisfaction, 27–32 = high satisfaction). The CSQ‐8 has been shown to have high internal consistency (α = 0.93) and is considered an ideal brief measure of client satisfaction (Attkisson and Zwick 1982).

##### 2.3.3.4 Use of Mental Health Services

Service users were asked to estimate the frequency of their use of community crisis services as well as the number of times they had been admitted to a psychiatric unit for mental health concerns while involved in the EPI service.

#### 2.3.4 Objective 5

Participants were given the opportunity to complete two open‐ended questions to gather additional feedback regarding their unique information needs: (1) *Are there any other types of information about treatments for psychosis that you think are important?* and (2) *Are there any other sources of information that you would find helpful?*

### 2.4 Data Analysis

#### 2.4.1 Objectives 1–4: Quantitative Analyses

Data were cleaned and analysed using the Statistical Package for Social Sciences (SPSS) Version 28. Prior to conducting analyses, all responses were reviewed for eligibility and completeness. Participants who completed the first 30% or less of the survey were removed as no outcome data would have been collected. Following, the data were visually reviewed for outliers using box plots. In total, five outliers were identified across several variables and were retained in the analysis. Responses of ‘prefer not to answer’ were not included in total scores for each measure and instead were only considered within descriptive analyses. Missing data ranged from 0% to 5.3% across MHL, information needs, stress, client satisfaction, attitudes towards psychological treatment, information needs, and items. Listwise deletion was employed as less than 10% of data was missing (Bennett 2001). Valid percentages are reported for any analyses with missing data.

Descriptive statistics and 95% confidence intervals were calculated for each MHL and information preference scale item. Independent samples *t*‐tests and Mann–Whitney *U*‐tests were used to assess differences in MHL total scores, information needs, and treatment outcomes between participant groups. Mann–Whitney *U*‐tests were used when assumptions of the parametric *t*‐test were violated. Additionally, linear regressions were computed to determine whether duration of EPI involvement was related to higher MHL and lower information needs (i.e., total information content, amount, source and format scores) among service users. A combination of linear and logistic regressions were used to determine whether MHL was a significant predictor of treatment outcomes. Assumptions of linear and logistic regression were assessed a priori.

#### 2.4.2 Objective 5: Qualitative Analysis

The open‐ended text responses were analysed by the first author (C.M.) using qualitative conventional content analysis (Hsieh and Shannon 2005). The responses were analysed in the order in which they appeared in the survey. The first author initially reviewed the responses word‐by‐word to identify key terms, took notes, and assigned preliminary code names that were reflective of emerging themes in the text. Initial codes were revised as needed (e.g., combining or discarding themes) and organised into categories. A coding journal was maintained to enhance the transparency of the analysis (Tracy 2010). The authors reviewed and finalised the names of each code. The authors included clinical psychologists and a graduate student in clinical psychology, each of whom brought a broad range of personal and professional experience to the analysis.

## 3 Results

Overall, *n* = 57 service users and *n* = 43 support persons completed the survey, which was lower than the number desired based on the power analysis. Participants' data were removed if they did not meet the inclusion criteria (*n* = 2) or if they completed 30% or less of the survey (*n* = 3). The final sample size (*n* = 95) included 53 service users and 42 support persons; this latter group was made up of parents (*n* = 34, 81%), siblings (*n* = 4, 9.5%) and romantic partners (*n* = 4, 9.5%). The average age of service users and support persons was 24.08 (SD = 4.02) and 48.32 (SD = 11.74) years old, respectively. The majority of the service user sample was male (*n* = 33, 64.7%), while the majority of the support person sample was female (*n* = 35, 83.3%). A large majority of both participant groups had a grade 12 education (*n* = 44, 83% of service users; *n* = 35, 83.3% of support persons) and self‐identified as being Black, Indigenous, or People of Colour (*n* = 38, 80.9% of services users, *n* = 22, 52.4% of support persons) compared to White (*n* = 9, 19.1% of services users, *n* = 20, 47.6% of support persons). Please refer to Murphy et al. (2025) for a more detailed analysis of the participant demographics.

### 3.1 Objective 1

#### 3.1.1 MHL

In total, 67.9% of service users (*n* = 36) endorsed having received psychoeducation on psychosis, treatment and recovery during their involvement with the EPI service. Of those who did not endorse having received psychoeducation, more than half (*n* = 9, 52%) were fairly new to the service (i.e., involved for ≤ 4 months). Table 1 illustrates service users' and support persons' responses on the MHL Scales. Most of the service users and support persons reported a moderate to very good understanding of the term ‘psychosis’ and its relationship to substance use. On the other hand, service users were less likely to report a good understanding of the terms, ‘positive’ and ‘negative’ symptoms, and even fewer respondents from both participant groups reported a good understanding of the stress‐vulnerability model. While the majority of services users and support persons (albeit to a lesser extent) felt they had a very good understanding of the EPI services and their respective roles in the treatment process, they were less familiar with community psychosis resources and with the local regional health authority's system. Finally, both participant groups tended to disagree with common stereotypes associated with the condition.

**TABLE 1:** Mental health literacy of psychosis among service users and support persons.

Item description	Service users					Support persons				
	Poor (%)	Moderate (%)	Very good (%)	Prefer not to respond (%)	Mean item rating [95% CI]	Poor (%)	Moderate (%)	Very good (%)	Prefer not to respond (%)	Mean item rating [95% CI]
Understanding of psychosis										
Term psychosis	2 (3.8)	23 (43.4)	27 (50.9)	1 (1.9)	5.56 [5.04, 6.08]	1 (2.5)	14 (35.0)	24 (60.0)	1 (2.5)	5.95 [5.38, 6.51]
Term ‘positive symptoms’	7 (13.2)	22 (41.5)	23 (43.4)	1 (1.9)	5.17 [4.56, 5.78]	9 (22.5)	11 (27.5)	20 (50.0)	0 (0.0)	5.08 [4.34, 5.82]
Term ‘negative symptoms’	7 (13.2)	21 (39.6)	23 (43.4)	2 (3.8)	5.15 [4.51, 5.78]	7 (17.5)	12 (30.0)	21 (52.5)	0 (0.0)	5.19 [4.47, 5.90]
Stress‐vulnerability model	14 (26.4)	25 (47.2)	12 (22.6)	2 (3.8)	3.90 [3.18, 4.62]	16 (41.0)	12 (30.8)	11 (28.2)	0 (0.0)	3.65 [2.72, 4.58]
Relationship between substance use and psychosis	3 (5.7)	15 (28.3)	33 (62.3)	2 (3.8)	5.77 [5.13, 6.41]	8 (20.0)	10 (25.0)	22 (55.0)	0 (0.0)	5.38 [4.58, 6.17]
Understanding of psychosis resources										
Community psychosis resources	11 (21.2)	18 (34.6)	21 (40.4)	2 (3.8)	4.60 [3.87, 5.34]	10 (25.0)	15 (37.5)	15 (27.5)	0 (0.0)	4.62 [3.84, 5.40]
Local regional health authority system	11 (20.8)	25 (47.2)	15 (28.3)	2 (3.8)	4.52 [3.91, 5.13]	9 (22.5)	15 (37.5)	16 (40.0)	0 (0.0)	4.46 [3.63, 5.29]
EPI clinic services	1 (1.9)	15 (28.3)	35 (66.0)	2 (3.8)	6.27 [5.83, 6.71]	3 (7.5)	15 (37.5)	22 (55.0)	0 (0.0)	5.49 [4.77, 6.20]

Item description	Not at all (%)	Moderately (%)	Completely (%)	Prefer not to respond (%)	Mean item rating [95% CI]	Not at all (%)	Moderately (%)	Completely (%)	Prefer not to respond (%)	Mean item rating [95% CI]
Understanding of role in treatment										
Understand role in own recovery process^a^	0 (0.0)	16 (30.8)	34 (65.4)	2 (3.8)	6.50 [6.07, 6.93]	—	—	—	—	—
Understand role of family within treatment^a^	3 (5.7)	21 (39.6)	26 (49.1)	3 (5.7)	5.75 [5.20, 6.30]	—	—	—	—	—
Understand role within loved one's treatment^b^	—	—	—	—	—	1 (2.5)	10 (25.0)	29 (72.5)	0 (0.0)	6.32 [5.80, 6.85]

Item description	Not at all agree (%)	Moderately agree (%)	Completely agree (%)	Prefer not to respond (%)	Mean item rating [95% CI]	Not at all agree (%)	Moderately agree (%)	Completely agree (%)	Prefer not to respond (%)	Mean item rating [95% CI]
Attitudes toward psychosis										
People with psychosis are violent and dangerous^c^	39 (73.6)	1 (1.9)	1 (1.9)	2 (3.8)	5.46 [4.95, 5.96]	24 (61.5)	12 (30.8)	2 (5.1)	1 (2.6)	4.89 [4.29, 5.49]
Psychosis is the result of personal weakness^c^	39 (73.6)	11 (20.8)	1 (1.9)	2 (3.8)	5.58 [4.96, 6.20]	24 (61.5)	12 (30.8)	2 (5.1)	1 (2.6)	5.78 [5.13, 6.44]
Psychosis is treatable	1 (1.9)	5 (9.4)	45 (84.9)	2 (3.8)	7.10 [6.70, 7.51]	0 (0.0)	7 (17.5)	33 (82.5)	0 (0.0)	6.89 [6.47, 7.31]

*Note:* Valid percentages reported for items with missing data.

^a^ Only service users were asked to respond to this item.

^b^ Only support persons were asked to respond to this item.

^c^ Denotes reverse coded item. Mean item scores have been reverse coded.

#### 3.1.2 Information Needs

Most service users (*n* = 41, 77.4%) and support persons (*n* = 35, 85.4%) reported using the Internet to search for information about psychosis in the past 12 months. Both groups reported being moderately familiar (*n* = 24, 45.3%; *n* = 19, 46.3%, respectively) or very familiar (*n* = 15, 28.3%; *n* = 15, 36.6%, respectively) with the types of help available for psychosis.

##### 3.1.2.1 Preferred Information Resources

Table 2 illustrates service users' and support persons' ratings of the helpfulness of various psychosis resources. The majority of service users preferred in‐person meetings with their healthcare provider and were most willing to engage in psychological and pharmacological treatment as well as peer support. Support persons expressed a willingness to access a greater variety of resources (e.g., family therapy, psychoeducation group, website, self‐help books, etc.).

**TABLE 2:** Helpfulness of various psychosis resources.

Type of resource	Service users					Support persons				
	Not at all helpful (%)	Moderately helpful (%)	Very helpful (%)	Prefer not to respond (%)	Mean item rating [95% CI]	Not at all helpful (%)	Moderately helpful (%)	Very helpful (%)	Prefer not to respond (%)	Mean item rating [95% CI]
Self‐help book	5 (9.6)	14 (26.9)	30 (57.7)	3 (5.8)	5.82 [5.16, 6.47]	4 (10.0)	7 (17.5)	29 (72.5)	0 (0.0)	6.21 [5.61, 6.80]
Self‐help website	4 (7.7)	17 (32.7)	28 (53.8)	3 (5.8)	5.78 [5.16, 6.39]	1 (2.5)	7 (17.5)	31 (77.5)	1 (2.5)	6.21 [5.67, 6.74]
Telephone conversation with healthcare provider	6 (11.3)	8 (15.1)	36 (67.9)	3 (5.7)	5.88 [5.20, 6.55]	2 (5.0)	5 (12.5)	33 (82.5)	0 (0.0)	6.69 [6.08, 7.31]
In‐person meeting with healthcare provider	4 (7.5)	5 (9.4)	41 (77.4)	3 (5.7)	6.57 [5.99, 7.15]	0 (0.0)	5 (12.5)	35 (87.5)	0 (0.0)	7.23 [6.82, 7.64]
Education group^a^	5 (9.6)	16 (30.8)	29 (55.8)	2 (3.8)	5.53 [4.87, 6.19]	1 (2.5)	5 (12.5)	34 (85.0)	0 (0.0)	6.92 [6.49, 7.36]
Discussion with an individual who has coped with psychosis^b^	3 (5.7)	13 (24.5)	35 (66.0)	2 (3.8)	6.24 [5.65, 6.83]	—	—	—	—	—
Taking medication recommended by psychiatrist^b^	2 (3.8)	12 (22.6)	37 (69.8)	2 (3.8)	6.54 [6.00, 7.08]	—	—	—	—	—
Receiving psychological treatment with a psychologist^b^	3 (5.7)	8 (15.1)	39 (73.6)	3 (5.7)	6.70 [6.15, 7.25]	—	—	—	—	—
Talking to another support person who has been involved with EPPIS^c^	—	—	—	—	—	0 (0.0)	9 (22.5)	31 (77.5)	0 (0.0)	6.82 [6.38, 7.27]
An appointment with a clinician with experience in family/relationship therapy^c^	—	—	—	—	—	0 (0.0)	2 (5.0)	38 (95.0)	0 (0.0)	7.50 [7.20, 7.80]

*Note:* Valid percentages reported for items with missing data.

^a^ Service users were asked about attending an education group with other young persons involved with EPPIS. Support persons were asked about attending an education group with other persons who know of an individual involved with EPPIS.

^b^ Only service users were asked to respond to this item.

^c^ Only support persons were asked to respond to this item.

##### 3.1.2.2 Type of Information Preferences

Table 3 illustrates information content preferences across service users and support persons. While support persons felt it was very important to receive information about all types of treatment for psychosis, service users were most interested in receiving information about counselling or psychological treatment options. Both service users and support persons felt it was important to receive a great deal of information about treatment (e.g., process, goals, effectiveness, consequences of discontinuing, pros/cons) and about the profession/training and experience of their health care professionals.

**TABLE 3:** Types of preferred information for psychosis.

Information content item	Service users					Support persons				
	Not at all important (%)	Moderately important (%)	Very important (%)	Prefer not to respond (%)	Mean item rating [95% CI]	Not at all important (%)	Moderately important (%)	Very important (%)	Prefer not to respond (%)	Mean item rating [95% CI]
All types of treatment	1 (1.9)	19 (35.8)	30 (56.6)	3 (5.7)	6.08 [5.56, 6.61]	0 (0.0)	0 (0.0)	37 (94.9)	2 (5.1)	6.80 [6.24, 7.36]
Medication treatments	1 (1.9)	18 (34.0)	32 (60.4)	2 (3.8)	6.13 [5.59, 6.66]	0 (0.0)	3 (7.5)	34 (85.0)	3 (7.5)	6.30 [5.62, 6.98]
Counselling or psychological treatments	2 (3.8)	9 (17.0)	40 (75.5)	2 (3.8)	6.44 [5.95, 6.93]	0 (0.0)	4 (10.0)	35 (87.5)	1 (2.5)	6.50 [5.80, 7.20]
Self‐help treatments	5 (9.4)	20 (37.7)	28 (52.8)	0 (0.0)	5.48 [4.89, 6.07]	1 (2.5)	17 (42.5)	21 (52.5)	1 (2.5)	4.60 [4.00, 5.20]
Alternative treatments	6 (11.3)	18 (34.0)	29 (54.7)	0 (0.0)	5.33 [4.69, 5.98]	2 (5.0)	12 (30.0)	24 (60.0)	2 (5.0)	5.10 [3.91, 6.29]
Role in treatment	1 (1.9)	14 (26.9)	35 (66.0)	2 (3.8)	6.29 [5.78, 6.80]	0 (0.0)	2 (5.0)	36 (90.0)	2 (5.0)	6.70 [5.94, 7.46]
Financial cost of treatment	10 (18.9)	11 (20.8)	30 (56.6)	2 (3.8)	5.44 [4.63, 6.24]	4 (10.3)	8 (20.5)	25 (64.1)	2 (5.1)	5.50 [4.10, 6.90]
The cost of treatment to the healthcare system	13 (25.0)	16 (30.8)	21 (40.4)	2 (3.8)	4.58 [3.77, 5.40]	7 (17.9)	13 (33.3)	15 (38.5)	4 (10.3)	4.80 [3.59, 6.01]
Effectiveness or success of treatment	1 (1.9)	8 (15.1)	43 (81.1)	1 (1.9)	6.85 [6.43, 7.28]	0 (0.0)	3 (7.5)	36 (90.0)	1 (2.5)	7.00 [6.33, 7.67]
How the treatment works	2 (3.8)	10 (18.9)	41 (77.4)	0 (0.0)	6.73 [6.29, 7.17]	0 (0.0)	1 (2.5)	39 (97.5)	0 (0.0)	7.10 [6.57, 7.63]
Goal or outcome of the treatment	0 (0.0)	6 (11.3)	46 (86.8)	1 (1.9)	6.88 [6.53, 7.22]	0 (0.0)	1 (2.5)	39 (97.5)	0 (0.0)	7.20 [6.64, 7.76]
How long for the treatment to reduce results	3 (5.7)	9 (17.0)	39 (73.6)	2 (3.8)	6.42 [5.87, 6.97]	2 (5.0)	6 (15.0)	32 (80.0)	0 (0.0)	6.10 [5.18, 7.02]
How long the treatment continues	2 (3.8)	11 (20.8)	39 (73.6)	1 (1.9)	6.54 [6.05, 7.04]	0 (0.0)	5 (12.5)	34 (85.0)	1 (2.5)	6.40 [5.50, 7.30]
What happens when treatment stops	4 (7.5)	7 (13.2)	41 (77.4)	1 (1.9)	6.56 [6.00, 7.13]	1 (2.5)	0 (0.0)	38 (95.0)	1 (2.5)	7.10 [6.47, 7.73]
Common side effects of treatment	4 (7.5)	10 (18.9)	38 (71.7)	1 (1.9)	6.23 [5.61, 6.85]	0 (0.0)	0 (0.0)	11 (28.9)	27 (71.1)	7.70 [7.35, 8.05]
Serious side effects of treatment	3 (5.7)	8 (15.1)	40 (75.5)	2 (3.8)	6.75 [6.20, 7.30]	0 (0.0)	1 (2.5)	39 (97.5)	0 (0.0)	7.20 [6.75, 7.65]
Pros and cons of each treatment	2 (3.8)	10 (19.2)	39 (75.0)	1 (1.9)	6.52 [5.97, 7.08]	0 (0.0)	1 (2.5)	39 (97.5)	0 (0.0)	6.70 [6.35, 7.05]
Training and profession of healthcare provider	0 (0.0)	10 (18.9)	41 (77.4)	2 (3.8)	6.67 [6.20, 7.13]	0 (0.0)	2 (5.0)	38 (95.0)	0 (0.0)	6.90 [6.37, 7.43]
Healthcare provider's experiences treating these problems	0 (0.0)	1 (18.9)	40 (75.5)	3 (5.7)	6.67 [6.21, 7.12]	0 (0.0)	0 (0.0)	39 (100.0)	0 (0.0)	6.90 [6.37, 7.43]
Waiting period before treatment starts	3 (5.8)	10 (19.2)	37 (71.2)	2 (3.8)	6.27 [5.72, 6.82]	0 (0.0)	3 (7.7)	36 (92.3)	0 (0.0)	6.40 [5.37, 7.42]
Where treatment will take place	3 (5.8)	11 (21.2)	35 (67.3)	3 (5.8)	6.17 [5.56, 6.78]	2 (5.3)	9 (23.7)	27 (71.1)	0 (0.0)	5.90 [5.11, 6.69]
Amount of time required to participate in treatment	1 (1.9)	11 (21.2)	37 (71.2)	3 (5.8)	6.48 [5.96, 7.00]	1 (2.6)	10 (25.6)	28 (71.8)	0 (0.0)	5.55 [4.89, 6.11]
Time of day treatment would be scheduled	2 (3.8)	12 (23.1)	34 (65.4)	4 (7.7)	6.23 [5.65, 6.81]	3 (7.7)	14 (35.9)	22 (56.4)	0 (0.0)	5.60 [4.91, 6.29]
Treatment options recommended by a healthcare provider	2 (3.8)	8 (15.4)	38 (73.1)	4 (7.7)	6.73 [6.16, 7.29]	0 (0.0)	1 (2.6)	28 (97.4)	0 (0.0)	6.70 [6.11, 7.29]

*Note:* Valid percentages are reported for any items with missing data.

##### 3.1.2.3 Source of Information Preferences

Table 4 highlights sources of information preferences across service users and support persons. Service users were most likely to seek information about psychosis from a healthcare provider specialising in mental health, their primary care provider, or a community mental health organisation. They were least likely to seek information from a religious leader or community Elder. In contrast, support persons were most likely to seek information from a religious leader or community Elder and least likely to seek information from a spouse, a community mental health organisation, or a primary care provider.

**TABLE 4:** Preferred sources of information among service users and support persons.

Source of information	Service users					Support persons				
	Not at all likely (%)	Moderately likely (%)	Very likely (%)	Prefer not to respond (%)	Mean item rating [95% CI]	Not at all likely (%)	Moderately likely (%)	Very likely (%)	Prefer not to respond (%)	Mean item rating [95% CI]
A romantic partner/spouse	10 (18.9)	11 (20.8)	29 (54.7)	3 (5.7)	5.44 [4.65, 6.23]	4 (10.0)	14 (35.0)	19 (47.5)	3 (7.5)	5.51 [4.74, 6.29]
One of your parents^a^	12 (22.6)	10 (18.9)	28 (52.8)	3 (5.7)	5.30 [4.52, 6.08]	—	—	—	—	—
A family member	10 (18.9)	15 (28.3)	25 (47.2)	3 (5.7)	5.08 [4.36, 5.80]	5 (12.5)	10 (25.0)	24 (60.0)	1 (2.5)	5.87 [5.18, 6.56]
A friend	10 (18.9)	15 (28.3)	25 (47.2)	3 (5.7)	5.16 [4.46, 5.86]	5 (12.5)	10 (25.0)	24 (60.0)	1 (2.5)	5.87 [5.18, 6.56]
Someone from a community mental health organisation	3 (5.7)	7 (13.2)	41 (77.4)	2 (3.8)	6.34 [5.79, 6.89]	4 (10.0)	16 (40.0)	18 (45.0)	2 (5.0)	5.08 [4.35, 5.81]
Religious leader or community elder	15 (28.3)	18 (34.0)	18 (34.0)	2 (3.8)	4.22 [3.46, 4.98]	0 (0.0)	6 (15.0)	33 (82.5)	1 (2.5)	7.03 [6.63, 7.42]
Primary care provider	3 (5.7)	6 (11.3)	42 (79.2)	2 (3.8)	6.46 [5.92, 7.00]	18 (45.0)	8 (20.0)	12 (30.0)	2 (5.0)	3.58 [2.58, 4.58]
Healthcare provider specialising in mental health	3 (5.7)	3 (5.7)	45 (84.9)	2 (3.8)	6.90 [6.35, 7.45]	3 (7.5)	7 (17.5)	29 (72.5)	1 (2.5)	6.41 [5.74, 7.08]

*Note:* Valid percentages are reported for any items with missing data.

^a^ Only service users were asked to respond to this item.

##### 3.1.2.4 Format of Information Preferences

Table 5 illustrates service user and support persons' responses on their preferred format of information. Both groups preferred to receive information through a discussion with a medical doctor; in addition, service users preferred to speak with a mental health clinician, and support persons preferred written information.

**TABLE 5:** Preferred information formats among service users and support persons.

Format of information	Service users					Support persons				
	Not at all preferred (%)	Moderately preferred (%)	Very much preferred (%)	Prefer not to respond (%)	Mean item rating [95% CI]	Not at all preferred (%)	Moderately preferred (%)	Very much preferred (%)	Prefer not to respond (%)	Mean item rating [95% CI]
Written form (e.g., information sheet or booklet)	4 (7.5)	19 (35.8)	27 (50.9)	3 (5.7)	5.64 [5.06, 6.22]	2 (5.0)	6 (15.0)	32 (80.0)	0 (0.0)	6.09 [5.34, 6.84]
Discussion with a medical doctor	1 (1.9)	10 (18.9)	39 (73.6)	3 (5.7)	6.56 [6.11, 7.01]	1 (2.5)	6 (15.0)	33 (82.5)	0 (0.0)	5.68 [5.11, 6.25]
Discussion with a mental health clinician	2 (3.8)	9 (17.0)	39 (73.6)	3 (5.7)	6.62 [6.13, 7.10]	0 (0.0)	3 (7.5)	19 (47.5)	18 (45.0)	6.73 [6.25, 7.20]
Video on the internet	6 (11.3)	15 (28.3)	30 (56.6)	2 (3.8)	5.60 [4.95, 6.25]	4 (10.0)	9 (22.5)	27 (67.5)	0 (0.0)	5.32 [4.45, 6.19]
Recommended internet website	5 (9.4)	14 (26.4)	31 (58.5)	3 (5.7)	5.78 [5.20, 6.36]	2 (5.0)	10 (25.0)	28 (70.0)	0 (0.0)	5.64 [4.82, 6.45]

*Note:* Valid percentages are reported for any items with missing data.

##### 3.1.2.5 Amount of Information Preferences

Table 6 illustrates service users' and support persons' preferred amount of information. Across both groups, there was a broad range of preferences for medication, psychological treatments, self‐help approaches, and alternative treatments, while most preferred 2–6 pages of information; a notable minority preferred 10 pages.

**TABLE 6:** Amount of information preferences among service users and support persons.

Type of information	0 pages (%)	2 pages (%)	4 pages (%)	6 pages (%)	8 pages (%)	10 pages (%)	Prefer not to respond (%)	Mean item rating [95% CI]
Medication treatment								
Service user	1 (1.9)	11 (20.8)	11 (20.8)	13 (24.5)	2 (3.8)	8 (15.1)	7 (13.2)	3.13 [2.65, 3.62]
Support persons	1 (2.6)	7 (17.9)	8 (20.5)	7 (17.9)	2 (5.1)	9 (23.1)	5 (12.8)	3.20 [2.62, 3.78]
Counselling or psychological treatments								
Service user	0 (0.0)	14 (26.4)	10 (18.9)	7 (13.2)	6 (11.3)	9 (17.0)	7 (13.2)	3.13 [2.63, 3.63]
Support persons	1 (2.6)	6 (15.4)	7 (17.9)	9 (23.1)	3 (7.7)	9 (23.1)	4 (10.3)	3.23 [2.67, 3.78]
Self‐help approaches (e.g., books or Internet‐based self‐help programmes)								
Service user	2 (3.8)	13 (24.5)	9 (17.0)	6 (11.3)	4 (7.5)	13 (24.5)	6 (11.3)	3.15 [2.62, 3.68]
Support persons	1 (2.6)	9 (23.1)	8 (20.5)	5 (12.8)	2 (5.1)	12 (30.8)	2 (5.1)	3.00 [2.42, 3.58]
Alternative treatments								
Service user	2 (3.8)	13 (25.0)	13 (25.0)	6 (11.5)	2 (3.8)	9 (17.3)	7 (13.5)	2.96 [2.44, 3.49]
Support persons	0 (0.0)	15 (38.5)	6 (15.4)	6 (15.4)	0 (0.0)	9 (23.1)	3 (7.7)	2.70 [2.11, 3.29]

*Note:* Valid percentages reported for items with missing data.

### 3.2 Objective 2

#### 3.2.1 Differences in MHL and Information Needs Between Groups

Table 7 illustrates the results of *t*‐tests comparing MHL and information needs between groups. An independent samples *t*‐test indicated that MHL did not significantly differ between service users and support persons. Likewise, Mann–Whitney U‐tests indicated that there were no significant differences in information content, amount, source, or format needs across groups.

**TABLE 7:** Differences in mental health literacy and information needs across groups.

	Service user, *M* (SD)	Support person, *M* (SD)	*T* or *U*	Degrees of freedom or *N*	*p*
Mental health literacy	65.58 (15.44)	63.70 (16.29)	0.54	83	0.59
Information content^a^	150.46 (33.28)	152.70 (13.09)	229.00	58	0.82
Information source	41.25 (8.76)	39.58 (9.91)	0.82	82	0.42
Information amount	12.23 (6.92)	12.38 (6.15)	−0.11	89	0.91
Information format^a^	30.20 (7.83)	29.45 (6.20)	506.50	72	0.59

*Note:* Total scores are displayed for each measure. Only items that were shown to both service users and support persons have been included in these total scores.

^a^ Mann Whitney *U*‐tests were conducted.

### 3.3 Objective 3

#### 3.3.1 Length of Treatment, MHL, and Information Needs

Service users received an average of 18.15 months of treatment (SD = 14.71). Results from a series of linear regressions indicated that length of treatment (months) significantly predicted total source of information preferences (*R* ^2^ = 0.37), *β* (0.12) = 0.33, 95% CI [0.09, 0.58], *p* = 0.009, but not service users' MHL or information content, format, or amount; see Table 8.

**TABLE 8:** Regression analyses: length of involvement with epi, mental health literacy, and information needs among service users.

Outcome variables	Estimate	SE	95% CI		*p*
			Lower limit	Upper limit	
Mental health literacy	0.11	0.16	−0.22	0.43	0.51
Information content needs	−0.19	0.33	−0.85	0.47	0.57
Information amount needs	0.02	0.07	−0.12	0.15	0.79
Information source needs	**0.33**	**0.12**	**0.09**	**0.58**	0.**01**
Information format needs	0.08	0.07	−0.07	0.23	0.29

*Note:* A simple linear regression model was constructed with length of EPI programme involvement (in months) entered as the independent variable. Significant values at *p* < 0.05 are indicated in bold type.

### 3.4 Objective 4

#### 3.4.1 Treatment Outcomes and MHL

Both service users and support persons reported moderate levels of stress (*M*_K6_ = 8.23, SD_K6_ = 5.84; *M*_K6_ = 5.71, SD_K6_ = 5.28, respectively). A Mann–Whitney *U*‐test indicated that service users had significantly higher levels of stress than support persons (*U* = 801.50, *p* = 0.04). Both service users and support persons reported positive attitudes toward seeking psychological treatment (*M*_ATSPPH‐SF_ = 21.60, SD_ATSPPH‐SF_ = 4.99; *M*_ATSSPH‐SF_ = 23.64, SD_ATSSPH‐SF_ = 4.28, respectively) and high treatment satisfaction (*M*_CSQ_ = 30.25, SD_CSQ_ = 3.31; *M*_CSQ_ = 29.08, SD_CSQ_ = 5.36, respectively). During their involvement with the EPI service, most service users had accessed community crisis services (*n* = 34, 64.2%) but had not required a psychiatric hospital admission (*n* = 36, 67.9%).

As reported in Table 9, a linear regression indicated that MHL scores significantly predicted service users' stress levels, *β* = −0.11 (0.05), 95% CI [−0.20, −0.02], *p* = 0.02, as well as client satisfaction as reported by both service users, *β* = 0.09 (0.03), 95% CI [0.04, 0.14], *p* = 0.002, and support persons, *β* = 0.18 (0.05), 95% CI [0.08, 0.28], *p* = < 0.001. A bivariate logistic regression model indicated that MHL did not significantly predict previous psychiatric hospitalisation nor access to crisis services; however, this latter predictor was approaching significance, *β* = −0.04, OR = 0.96, 95% CI [0.92, 1.00], *p* = 0.06.

**TABLE 9:** Regression analyses: mental health literacy and treatment outcomes.

Outcome variables	Estimate	SE	Odds ratio	95% CI		*p*
				Lower limit	Upper limit	
Stress (K6)^a^						
Service users	**−0.11**	**0.05**	—	**−0.20**	**−0.02**	**0.02**
Support persons	0.01	0.05	—	−0.10	0.12	0.81
Client satisfaction^a^						
Service users	**0.09**	**0.03**	—	**0.04**	**0.14**	**0.002**
Support persons	**0.18**	**0.05**	—	**0.08**	**0.28**	**< 0.001**
Attitudes towards psychological service use^a^						
Service users	0.07	0.04	—	−0.02	0.16	0.12
Support persons	0.07	0.05	—	−0.02	0.16	0.13
Number of psychiatric hospitalizations^b^						
Service users	−0.01	0.02	1.00	0.96	1.04	0.94
Use of crisis services^c^						
Service users	−0.04	0.02	0.96	0.92	1.00	0.06

*Note:* Significant values at *p* < 0.05 are indicated in bold type.

^a^ A simple linear regression model was constructed with mental health literacy entered as the independent variable.

^b^ A logistic regression was conducted using mental health literacy as the independent variable and past psychiatric hospitalizations (0 = none, 1 = at least 1 psychiatric hospitalisation) as the outcome variable.

^c^ A logistic regression model was conducted using mental health literacy as the independent variable and crisis service use (0 = no, 1 = yes) as the outcome variable.

### 3.5 Objective 5

#### 3.5.1 Additional Information Needs

In response to the question, “Are there any other types of information about treatments for psychosis that you think are important?” both service users and support persons included similar reflections. As such, the following main themes were developed with the experiences of both groups in mind: Diagnosis and Recovery; Treatment; and Healthy Functioning. See Table 10 to review written responses.

**TABLE 10:** Qualitative responses.

Theme	Participant	Participant group	Direct quote
Diagnosis and recovery	P79	Service user	I think psychosis is important most of the time
	P53	Service user	What [is] the [likelihood] for another psychosis episode
	P22	Service user	I do not have much knowledge about it
	P44	Service user	What the recovery path looks like
	P14	Service user	Rest is important
	P31	Service user	‘If you have psychosis, you are not crazy or selfish; you have a disease [that requires] help and treatment… to heal.’
	P61	Support person	Provision of confidential/general examples of cases of people navigating through psychosis to have more of a general idea of how to approach the young person going through psychosis
	P1	Support person	I would also like to know what we can do to support [the loved one]
	P67	Support person	Offer support for parents and caregivers [to learn] how they can help the family member with [psychosis]. This is a BIG missing piece of the puzzle
	P58	Support person	Correlation between drug use and psychosis. Why is the term ‘drug induced’ thrown around? Is there insight to drug induced versus genetics versus genetic disposition accelerated by drug use and so forth…
	P78	Support person	Provide more information on diagnosis
	P20	Support person	All information is important to me
	P12	Support person	Learning more about the illness schizophrenia and how it works for the person who has it and the person who is dealing with the person who has it
	P64	Support person	I also strongly believe [the loved one] has been misdiagnosed, [the loved one has] had three or four separate [diagnoses] now, which leads me to strongly believe [the loved one is] on the wrong medication. This could be why [the loved one is] in such a fog, that [the loved one] wakes up every morning looking and feeling hungover.
	P68	Support person	Preventative measures
Treatment	P5	Service user	More insight into the effects of antipsychotic medication and lasting impacts [the medication] may have
	P51	Service user	More information on psychosis medication
	P14	Service user	Keep up with the [medications]
	P13	Service user	I struggle with knowing how many prescription drugs I am on
	P45	Service user	Treatments for psychosis when the client is considering on taking a vacation.
	P64	Support person	Management of side effects
	P63	Support person	The programme my [loved one] is in has been wonderful. The medications are being adjusted and we are seeing and [the loved one] has been sharing with us that [the loved one's] symptoms are amplified. How does adjusting [medications] when [the medications] seemed to be working [factor] into [the loved one's] care?…
	P1	Support person	You mentioned medication, goals of the programme and treatment options. This is important information to me. I know very little about the medications available and I would like to know what the options are and what kind of side effects there are, taking into account that I am not a psychiatrist and I trust that [the psychiatrist] knows what is best for our [loved one].
Healthy functioning	P43	Service user	[Learn to] speak for yourself
	P44	Service user	Possible timelines for returning to work, how to know you are ready to begin returning to work
	P10	Service user	Less stress job. Doing something that doesn't require lot of mental energy.
	P62	Service user	Ear ringing
	P7	Support person	Nutrition, exercise, self‐care, social contacts
	P1	Support person	How to let go when [the loved one] needs more independence
Reliable sources	P31	Service user	Books in my mother‐language. Information from [T.V.] dramas
	P44	Service user	Books or self‐help [workbooks]
	P39	Service user	Maybe a learning portal for psychosis, but I don't think it's necessary
	P72	Service user	YouTube
	P46	Service user	Verbal information is helpful for me
	P37	Service user	Professional psychologist
	P73	Support person	Library reading lists
	P2	Support person	Recommended apps
	P68	Support person	Access to recommended reading
	P63	Support person	Private viewing or reading is my preference at this time.
	P12	Support person	One on one sessions
	P7	Support person	Family doctor
	P61	Support person	None that come to mind. Just direct contact with explanations would work.
	P64	Support person	Where to find reliable sources of information
Group programming	P52	Service user	Perhaps a group information session. I like groups in person!
	P28	Service user	I suggest getting people to participate in the group I was in since it helps explains our thinking [a lot]
	P44	Service user	I am at the beginning of my journey so haven't had group therapy options yet but I am looking forward to participating in them this fall
	P38	Service user	Suggesting the family and individual psychosis groups early on in treatment
	P68	Support person	Maybe small group discussions
	P1	Support person	I went to a session on families and mental health and the care programme. It was very helpful to watch the [PowerPoint] every week, listen to the presenter discuss strategies and provide information, and to listen to other people's stories.
	P12	Support person	Groups so that you don't feel alone in your psychosis
	P69	Support person	More activities
Peer support	P45	Service user	Experiences with clients within [EPPIS]
	P16	Service user	Patients who have recovered
	P66	Support person	[Are] there other moms out there to talk to when you just want to cry [and] feel [somewhat] not so alone knowing someone is going [through] the same thing with their own child
	P1	Support person	I understand that there will be a family support group in February. I am really looking forward to that. Our [loved one] has only been taking part for about a month but I feel this is a wonderful programme.

##### 3.5.1.1 Diagnosis and Recovery

Participants expressed a desire to learn more about the various diagnoses often associated with first episode psychosis, including the terms ‘schizophrenia’ (P12, support person) and ‘drug‐induced psychosis’ (P58, support person). One service user indicated that they did ‘not have much knowledge about [psychosis] (P22) despite their involvement in the EPI service. Another support person (P64) expressed frustration and confusion over the fact that their loved one has been given ‘3 or 4’ diagnostic labels, leading them to question the current diagnosis and treatment plan.

Participants were also interested in long‐term prognosis, such as ‘what the recovery path looks like’ (P4, service user), ‘the [likelihood] [of having] another psychotic episode’ (P53, service user), and ‘preventative measures’ (P68, support person). Support persons (P1, P12, P61) expressed a desire to learn effective communication strategies and other strategies to help the young person (P67).

Service users shared information they felt was important for their peers to know, such as the importance of rest (P14) and that ‘if you have psychosis, you are not crazy or selfish; you have a disease [that requires] help and treatment… to heal (P31).

##### 3.5.1.2 Treatment

Participants requested specific information related to medication. Indeed, P1 (support person) wrote,

You mentioned medication, goals of the programme, and treatment options. This is important information to me. I know very little about the medications available and I would like to know what the options are and what kind of side effects there are, taking into account that I am not a psychiatrist and I trust that [the psychiatrist] knows what is best for our [loved one].

Other questions related to the various types of antipsychotic medication available (P51; service user), potential side effects (P64, support person; P5, service user), how to manage medication during vacation (P45, service user), and reasons why a medication would be titrated down if the dosage appeared to reduce symptoms (P63, support person). P13 (service user) noted that they struggle to know about the different medications prescribed to them. P14 (service user) thought others should be made aware of the negative impact of medication nonadherence.

##### 3.5.1.3 Healthy Functioning

Support persons requested more information on physical exercise, nutrition, self‐care, and socialisation (P7, P69, P70). In wanting to best support their loved one, P1 asked how they can learn ‘to let go when [the loved one] needs more independence’, while P43 wanted to learn more assertive communication skills. P44 (service user) was curious about when they might return to the workforce, while P10 (service user) felt it was important for service users to consider low‐stress employment options.

#### 3.5.2 Additional Sources of Information

In response to the question ‘Are there any other sources of information that you would find helpful?’, participants' responses reflected the following themes: Reliable Sources; Group Programming; and Peer Support (see Table 10).

##### 3.5.2.1 Reliable Sources

Several participants requested written information, such as self‐help workbooks (P44, service user), literature available in a library (P73; P74, support persons), recommended readings (P68, support person), or books written in their primary language (P31, service user). Others wanted to receive information through recommended mobile apps (P2, support person), an online learning portal (P39, service user), or YouTube videos (P72, service user). While P63 (support person) noted that ‘private viewing or reading’ was important, others preferred oral discussions (P46, service user), including with a psychologist (P37, service user) and family physician (P7, support person).

##### 3.5.2.2 Group Programming

Several participants expressed an interest in attending group programming and activities (P52, service user; P69, support person; P44, service user), including those that allow for small group discussions (P68; support person). Indeed, P28 (service user) found groups helpful to better understand their thinking processes, while P12 (support person) noted that groups can help service users to not ‘feel alone in your psychosis.’ One support person (P1) who attended a family psychoeducation session shared that they found it ‘very helpful to watch the [PowerPoint] every week, listen to the presenters discuss strategies and provide information, and to listen to other people's stories.’ Furthermore, P38 (service user) recommended that both service users and support persons consider registering for the psychoeducation sessions early on in the recovery process.

##### 3.5.2.3 Peer Support

Both groups expressed interest in accessing peer‐support services. For example, service users wanted to learn about ‘experiences with clients within [EPI service]’ (P45) or from ‘patients who have recovered’ (P16). In a similar fashion, P66 (support person) wanted to know if there were other parents ‘to talk to when you just want to cry [and] feel… not so alone knowing someone is going [through] the same thing with their own child.’ P1 (support person) noted that they looked forward to attending community‐organised family peer support group.

## 4 Discussion

This is the first study to examine the MHL and information needs of service users involved in an EPI service and their designated support persons.

### 4.1 Objective 1

Both service users and support persons reported varying levels of MHL related to psychosis. For example, while the majority of participants had a moderate to good understanding of the term ‘psychosis’ and its relationship to substance use, there was more variability in their understanding of ‘positive’ and ‘negative’ symptoms. Furthermore, an even smaller number of participants felt they had a good understanding of the stress‐vulnerability model, community psychosis resources, and the local regional health authority. It was also interesting to note that support persons were less likely to report a good understanding of the EPI service compared to service users. Both groups identified a wide range of preferred information resources, types, and formats. One notable exception was the source from which they wanted to receive information; namely, while support persons indicated that they were most likely to seek information from a religious leader or community Elder, service users were the least likely to seek information from these sources.

While the majority of participants did not endorse stereotypical beliefs about psychosis, there were a minority of respondents from both participant groups who completely agreed with certain stigmatising beliefs. Stigma toward those with schizophrenia is associated with more negative beliefs compared to anxiety and depression (Pescosolido et al. 2021; Wood et al. 2014). Our findings support the call for continued anti‐stigma work and the implementation of additional strategies to address misconceptions about psychosis, including with those who are personally affected by the condition.

### 4.2 Objective 2

We did not find significant differences between service users and support persons in terms of their MHL and information needs. Both participant groups could benefit from access to additional psychoeducational materials and supports.

### 4.3 Objective 3

Among service users, longer involvement with the EPI service was significantly positively correlated with higher scores on total source of information preference scores; that is, being involved in our service for a longer period of time was associated with a greater openness to receiving information from a variety of sources. On the other hand, MHL was not correlated with duration of time involved in the service. While past research found that information needs persist after initial consultation or the initiation of pharmacological treatment (Addington et al. 2012), our study suggests that these information needs might actually persist well into the service user's involvement in a multi‐year EPI service.

### 4.4 Objective 4

Higher MHL was significantly positively correlated with treatment satisfaction among both participant groups. These results further extend prior MHL findings to first episode psychosis, namely that poor MHL is associated with decreased treatment satisfaction (Hall et al. 1988). Higher MHL was also significantly negatively associated with stress among service users. Given that stress is known to precipitate the initial onset and relapse of psychotic symptoms (Mondelli 2014), this latter finding is particularly of interest for service users; that is, a better understanding of psychosis is associated with lower levels of stress.

Although higher MHL had been found to reduce the likelihood of symptom relapse (Hall et al. 1988), this remains to be seen with first episode psychosis. While lower MHL was negatively correlated with the use of community crisis services, this association was only approaching significance. There was no association between MHL and the number of psychiatric hospital admissions. While these analyses may have lacked sufficient power (see *Limitations*), the findings could also reflect the effectiveness of EPI compared to standard community mental health care. The model of care facilitates the development of supportive and engaged alliances between the service user and their EPI team, allowing the team to intervene in a nimble and flexible manner should there be any signs of worsening psychotic and co‐morbid symptoms; in some cases, preventing the service user from requiring a psychiatric hospital admission or using crisis services. Future research should investigate whether MHL is related to other, more sensitive indicators of symptom relapse, such as symptom rating scales, frequency/duration of contact with the EPI team, or psychosocial functioning.

It is also notable that both groups of participants reported moderate levels of stress. This finding reflects the significant impact that first episode psychosis can have on the entire family unit, and validates the need for specific family supports beyond simply having a service user involved in an EPI service (Askey et al. 2009; Lucksted et al. 2018; Sin et al. 2005). Indeed, given that we found support persons have varying levels of MHL, high information needs, and moderate stress levels, the present research affirms the importance of providing additional support and family interventions to support persons, consistent with the best practice guidelines (Chien et al. 2019; International Early Psychosis Association Writing Group 2005; Lecomte et al. 2017; National Institute for Health and Clinical Excellence 2014; Norman et al. 2017).

### 4.5 Objective 5

When given the opportunity to identify unexplored information needs, many service users and support persons included themes related to diagnosis and recovery, treatment, and healthy functioning. Furthermore, both groups expressed a desire to access reliable sources, attend group programming, and connect with integrated peer supports.

### 4.6 Limitations

The study was launched during the COVID‐19 pandemic, when there were significant changes to the EPI service delivery model. Indeed, in‐person services were restricted, the focus of appointments was narrowed, and psychoeducational groups were strictly virtual. These changes likely resulted in disruptions to care delivery and psychoeducation within our pandemic cohort. The pandemic also created challenges with recruitment, leading to an underpowered study. We found that involving EPI clinicians in the recruitment process and providing immediate access to surveys during clinic visits resulted in higher levels of engagement compared to recruitment posters or mailed invitations alone.

This study used a convenience sample and correlational methodologies to enhance the ecological validity of MHL and information needs. The observed associations can generate hypotheses for randomised controlled studies that will more clearly delineate how EPI care delivery and psychoeducation affect MHL and information needs. Measures of insight and symptom rating scales could be included to account for confounding variables. Finally, the present study only recruited young adults with first episode psychosis; future research should investigate the MHL and information needs of adolescent minors.

### 4.7 Future Directions

International practice guidelines for first episode psychosis recommend the provision of intense and prolonged psychoeducational intervention during the initial management phase of illness (i.e., first 6 months of onset) (International Early Psychosis Association Writing Group 2005). Our findings suggests that it could be worthwhile to extend this intervention to the recovery phase as well. Service users and support persons could benefit from additional psychoeducation related to psychosis and related mental health supports and resources, including those topics that one might assume a participant involved in an EPI service would already be familiar with, such as clinical terminology, treatment information, and the training and experience of interprofessional team members. Service users expressed an interest in learning about psychological interventions, such as cognitive behaviour therapy for psychosis (CBTp) and cognitive remediation. In terms of clinical practice, clinicians should consider regular check‐ins with both service users and support persons to inquire about any unaddressed information needs throughout their time in the service. The results can inform EPI service improvements, such as tailoring psychoeducation to consider the content, modality, and the timing of delivery. Information should be designed and disseminated using a variety of formats and sources that are tailored to meet the individual needs of both service users and support persons.

In order to better address unmet information needs, focus groups with service users, support persons, and EPI team members were held to support knowledge translation activities and further evaluate informational sources. Based on this additional feedback, the EPI service has developed additional psychoeducational resources. To increase the awareness of psychosis and EPI in the general population, we are working to develop a programme website, informational brochures, and EPI signage to be posted in family practitioner offices, university counselling centres, and other relevant settings. We also have developed more tailored informational materials for service users and their support persons, including an information booklet and a lending library with self‐help books related to psychosis. These efforts will provide a variety of informational formats with a particular focus on developing written materials for support persons. We have implemented an evaluation of our manualised psychoeducational groups to assess the acceptability and effectiveness of these groups, as well as MHL and information needs. Finally, we have prepared a second manuscript (Murphy et al. 2025) outlining participant perspectives on the EPI service, including the impact of caregiving on support persons, what aspects of EPI have been helpful, and recommendations for programme improvement. These outcomes aim to improve direct patient care.

## 5 Conclusion

This is the first study to examine the MHL and information needs of both service users involved in an EPI service and their designated support persons. Findings indicate that higher MHL is correlated with reduced psychological distress in service users and higher treatment satisfaction among both participant groups. These results suggest that EPI programming and psychoeducation efforts should emphasise information about psychosis and available resources and continue to challenge stigmatising beliefs. It is also important to have multiple sources and formats that provide information tailored to the specific needs of both service users and their support persons throughout their involvement in the EPI service.

## Funding

This work was supported by Manitoba Medical Service Foundation, 8‐2020‐07 and the Winnipeg Foundation, 2020‐2583.

## Ethics Statement

This research has been approved by the Research Ethics Board at the University of Manitoba, Bannatyne campus (HS23765 [H2020:159]) and Shared Health Research Impact Committee (RI2020:031).

## Consent

All participants provided informed consent to participate. No minors participated in the research study.

## Conflicts of Interest

Colleen Murphy is a full member of the Canadian Consortium for Early Intervention in Psychosis and volunteers on the Education Committee. She is a member of the Canadian Network for Research in Schizophrenia and Psychoses and a member‐at‐large on the Steering Committee for the North American Cognitive Behavioural Therapy for psychosis Network. The other authors declare no conflicts of interest.

## Data Availability

The datasets generated and/or analyzed during the current study are not publicly available to protect the privacy of service users and their loved ones, but are available from the corresponding author upon reasonable request.
